# Informatic analysis of the pulmonary microecology in non-cystic fibrosis bronchiectasis at three different stages

**DOI:** 10.1515/biol-2022-0014

**Published:** 2022-02-28

**Authors:** Yuchao Wang, Ying Chen, Chao Wu, Xiaohong Yang

**Affiliations:** Graduate School, Xinjiang Medical University, 830001 Urumqi, China; Department of Respiratory and Critical Care Medicine, People’s Hospital of Xinjiang Uygur Autonomous Region, No. 91 Tianchi Road, Tianshan District, Urumqi 830001, China

**Keywords:** non-cystic fibrosis bronchiectasis, pulmonary microecology, informatics, genome sequencing, network dataset

## Abstract

This study explored the impact of pulmonary microecological changes on disease progression in non-cystic fibrosis bronchiectasis (nCFB). A careful search of the NCBI BioProject database revealed the 16S rRNA-based microbiological testing results of 441 pulmonary sputum samples from patients in the relatively stable (baseline), acute exacerbation, or recovery stage. After preliminary analysis and screening, we selected 152 samples for further analyses, including determination of the operational taxonomic unit (OTU) distribution at the phylum, class, order, family and genus levels, community structure, alpha diversity, beta diversity, microbial multivariables, correlations, and community structure after the abundances of intragroup samples were averaged. The recovery group showed significant differences in pulmonary microbiological changes (*P* < 0.05) compared with the other groups. There were 30 differentially abundant OTUs, with 27 and 7 at the genus and phylum levels, respectively. The Chao1 value of the recovery group was comparable to that of the baseline group, and the Shannon and Simpson values of the recovery group were the highest. Rhodococcus in Actinobacteria was positively correlated with Ochrobactrum in Firmicutes. The differences in pulmonary microecological changes at different nCFB stages may serve as a biologically predictive indicator of nCFB progression.

## 1 Introduction

Non-cystic fibrosis bronchiectasis (nCFB) is a chronic pulmonary disease characterized pathologically by permanent dilation and chronic inflammation of the bronchi and bronchioles and clinically by chronic progressive cough and hemoptysis dyspnoea and exacerbated recurrent infection [1]. It is the final common pathway through which various inflammatory, hereditary, autoimmune, developmental, and allergic diseases funnel [2,3,4,5,6]. Compared to patients without nCFB, those with nCFB have higher all-cause-, respiratory- and lung cancer-related mortalities. nCFB has brought a heavy economic burden to the medical system and is becoming a new-type chronic pandemic disease [7,8,9,10,11].

In patients with nCFB, core species exist in the pulmonary microecology, such as *Pseudomonas aeruginosa*, *Haemophilus influenzae,* and *Streptococcus pneumoniae*; these microorganisms may be associated with decreased pulmonary function and pathological reoccurrence and aggravation in these patients [12]. According to a cross-sectional study [13], pulmonary microecology is associated with disease progression in nCFB patients. This association is influenced by multiple mixed variables, particularly the disease phenotypes, antibiotic use, age, and sex of the patients; moreover, the effect of antibiotics on the pulmonary microecology of patients with nCFB varies according to the type, administration frequency, and duration of use of the antibiotics [13].

Based on the context mentioned above, we reanalyzed the raw 16S rRNA-based data regarding the pulmonary microecology in nCFB and investigated the progressive changes in the pulmonary microecology in patients with nCFB at different stages based on informatic analyses.

## 2 Materials and methods

### 2.1 Data collection

To perform the current longitudinal analysis, we performed a thorough search across the NCBI BioProject database (https://www.ncbi.nlm.nih.gov/bioproject/), with “bronchiectasis” as the keyword. A total of 42 results were obtained. Then, screening was performed according to the detailed information of each dataset: 11 datasets were removed due to their focus on a single disease stage, 16 were removed due to their focus on the association of single bacterial species (*Pseudomonas aeruginosa*, *Staphylococcus aureus*, Mycobacterium, and so on) with the disease, 2 were excluded due to the lack of raw data, and 9 were excluded due to being unsuitable for analysis (lack of specific grouping information, data platforms unsuitable for analysis, insufficient sample size, and so on). Finally, four datasets were selected.

### 2.2 Grouping

The four datasets were merged, and the involved patients were divided into the baseline group, the acute exacerbation group, and the recovery group. In the baseline group, slight aggravation of clinical symptoms was allowed, and antibiotic use had not occurred in the previous 30 days. In the exacerbation group, the diagnosis was made by specialists who also prescribed intravenous or oral antibiotics, and sputum was sampled before antibiotic use. The recovery group included patients who had terminated antibiotic use at least 1 month prior. The total number of selected samples was 454 from three refs [13–15]. For the sake of comparability among the samples in terms of different observational indices, 7 samples were excluded from the recovery group for weak correlation according to correlation analysis, and 18 and 186 were excluded from the exacerbation group and the baseline group, respectively, according to correlation analysis, clustering analysis, and principal component analysis. Eventually, the baseline group contained 60 cases, the exacerbation group contained 45 cases, and the recovery group included 48 cases.

The baseline data of the three groups are summarized in Table 1.

**Table 1:** Baseline data of the three groups

Index	Stratification	B (*n* = 60)	E (*n* = 45)	R (*n* = 48)	*P*
Age		58.25 ± 14.204	59.28 ± 10.710	58.09 ± 14.373	0.935
FEV1 (% predicted)		66.87 ± 23.985	63.36 ± 25.559	69.16 ± 24.344	0.636
BMI class (%)	Normal	29 (48.3)	22 (48.9)	31 (64.6)	0.722
	Obesity	3 (5)	6 (13.3)	4 (8.3)	
	Underweight	28 (46.7)	17 (37.8)	13 (27.1)	
Sex (%)	Female	38 (63.3)	34 (75.6)	35 (72.9)	0.839
	Male	22 (36.7)	11 (24.4)	13 (27.1)	
Cause (%)	PCD	8 (13.3)	4 (8.9)	7 (14.6)	0.36
	Immunodeficiency	1 (1.7)	1 (2.2)	0 (0.0)	
	Idiopathic	27 (45.0)	17 (37.8)	28 (58.3)	
	ABPA	5 (8.3)	11 (24.4)	7 (14.6)	
	Postinfection	18 (30.0)	12 (26.7)	6 (12.5)	
Smoking (%)	N	42 (70)	28 (62.2)	32 (66.7)	0.417
	Y	18 (30)	17 (37.8)	16 (33.3)	

Note: B, baseline; E, exacerbation; R, recovery; BMI, body mass index; ABPA, allergic bronchopulmonary aspergillosis; PCD, primary ciliary dyskinesia (Kartagener syndrome); FEV1, Forced Expiratory Volume In 1s.

### 2.3 Statistical analysis

**Figure 1:**
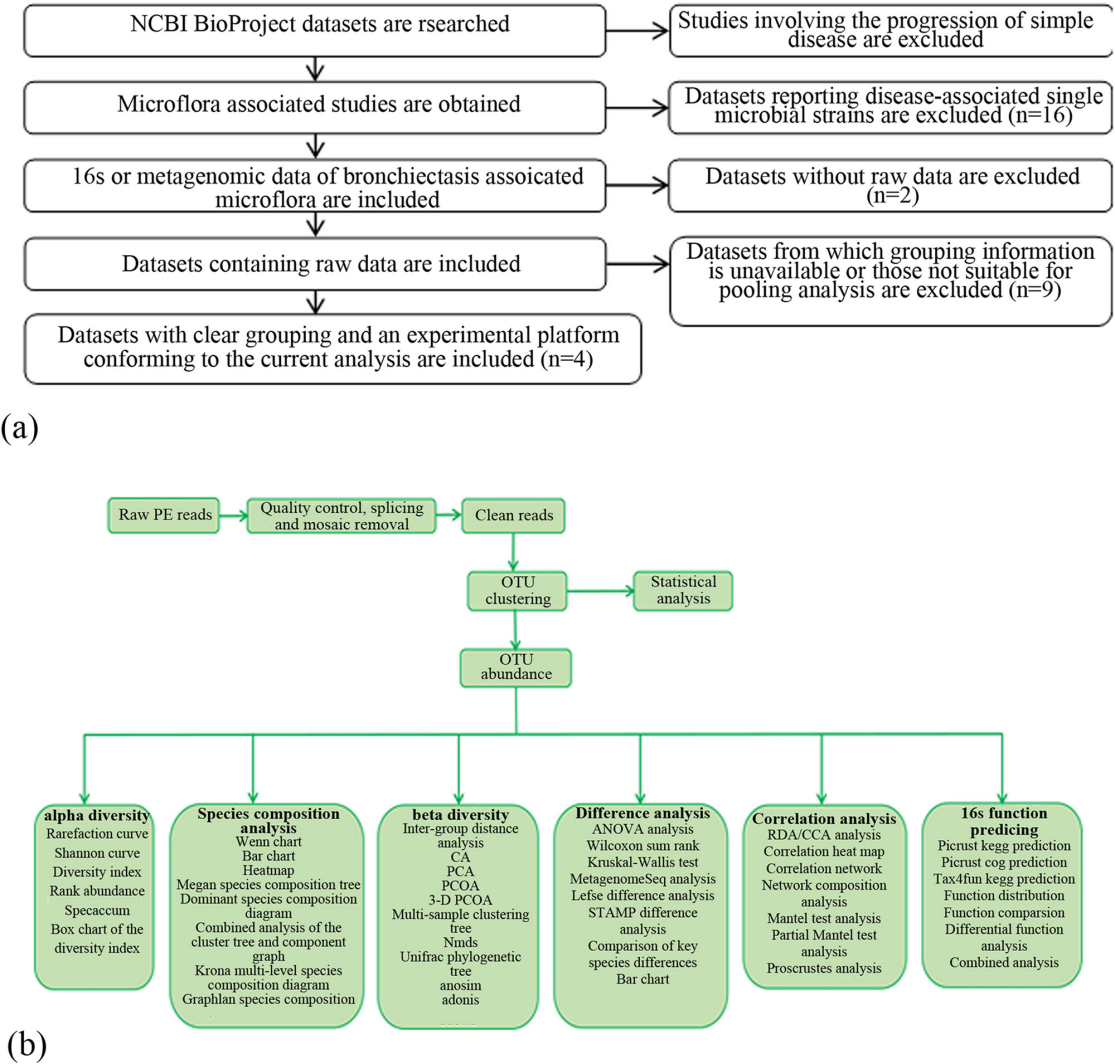
The flow diagram of the study design. (a) sample screening route; (b) informatic analysis route.

Vsearch (version 2.4.2) was used for operational taxonomic unit (OTU) classification [16]. After quality control, high-quality sequences (valid tags) were applied for OTU classification according to a sequence similarity threshold of 97%. The sequence with the maximum abundance in each OTU was taken as the representative sequence of the OTU. The obtained sequence was compared with the descriptions in the database and then annotated using the RDP classifier naive Bayesian classification algorithm [17] to obtain the annotation information of the corresponding OTU. According to the number of sequences for each OTU in each sample, an abundance matrix of the OTUs in each sample (otu_table.biom) was constructed. Based on sequence comparison, the phylogeny of the representative sequences of the OTUs was constructed with PyNAST (v0.1) software [18] and a phylogenetic tree file (rep_phylo.tre) was obtained. According to the principle of minimum depth, all the samples were randomly selected to obtain an OTU table (otu_table_even.biom). The number of tags in each sample that was integrated into the OTU table was calculated, and the abundance of each OTU in each sample was determined (Figure 1).

Data were analyzed using SPSS 25.0. For continuous variables with a normal distribution, analysis of variance (ANOVA) was used to compare among groups, and for those with abnormal distribution, nonparametric Kruskal–Wallis ANOVA was used. Count data are presented as the number (percentage) and were tested using the chi-square method. A difference with *P* < 0.05 was considered to be statistically significant.

## 3 Results

### 3.1 Flora structure

**Figure 2:**
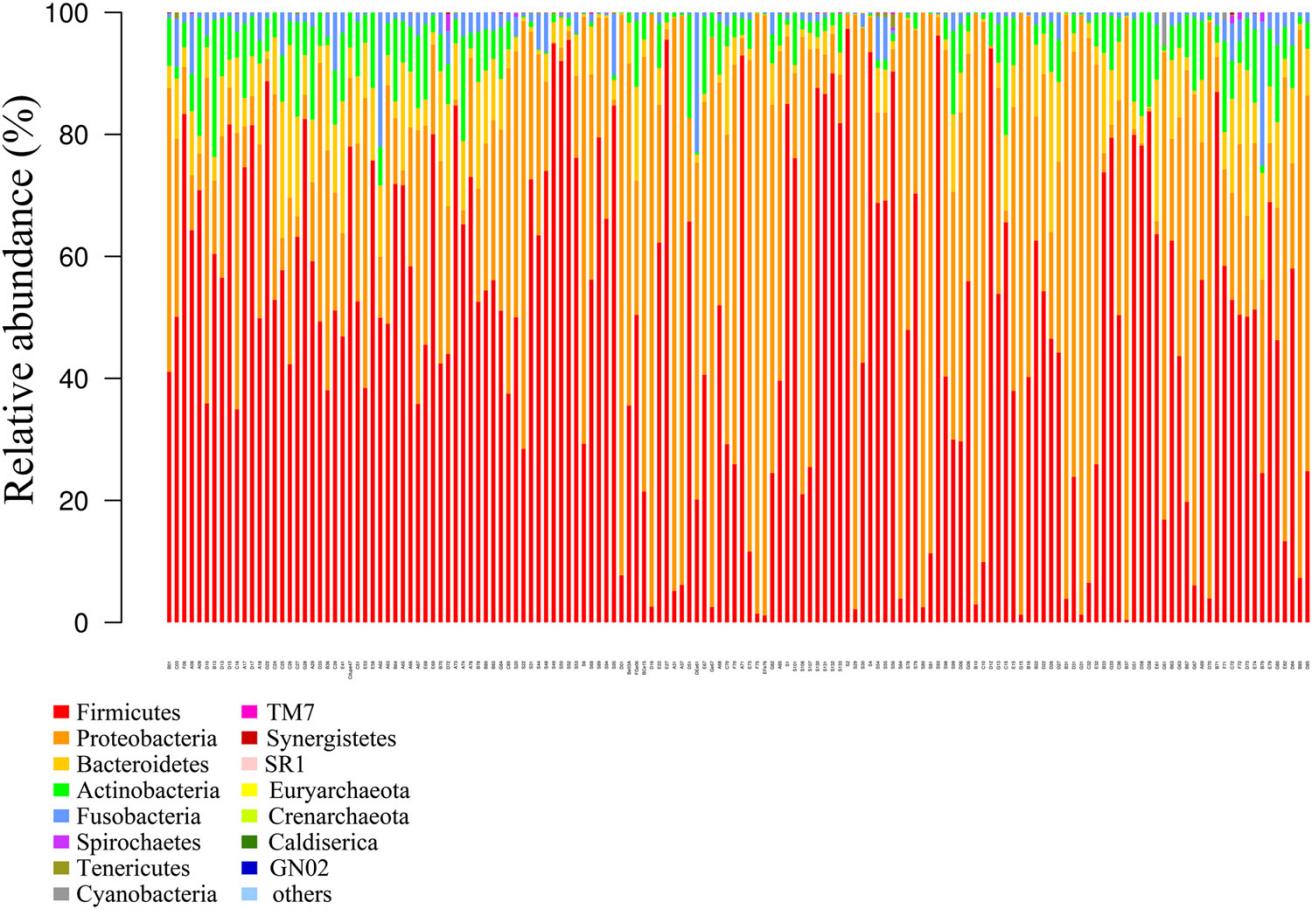
Histogram of the flora structure of the samples (top 15).

**Figure 3:**
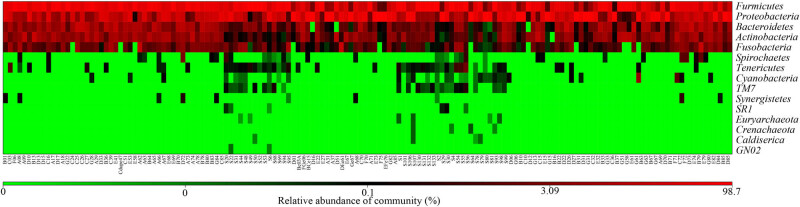
Heatmap based on relative abundances (top 15).

According to the OTU annotation outcomes, a number of the OTUs corresponded to the same genus or species. The classification outcomes were pooled, and the relative abundances of the samples at different levels were obtained (Figures 2 and 3). At the phylum level, the top ten microorganisms were Firmicutes, Proteobacteria, Bacteroidetes, Actinobacteria, Fusobacteria, Spirochaetes, Tenericutes, Cyanobacteria, TM7, and Synergistetes. At the genus level, the top ten microorganisms were Streptococcus, Pseudomonas, Haemophilus, Prevotella, Neisseria, Veillonella, Rothia, Granulicatella, Gemella, and Fusobacterium. Due to the lack of raw data in some samples, we did not perform analysis at the species level.

### 3.2 Alpha diversity

To compare the diversities among the samples, we normalized sampling depths before analysis. Alpha diversity analysis showed that the coverage of all the samples approached 1. The three groups showed significant differences in alpha diversity. The Chao1 value of the recovery group was close to that of the baseline group. In addition, the Shannon (3.74213) and Simpson (0.836411) values of the recovery group were the highest among the three groups. Compared to the baseline group, the recovery group did not show significant differences in terms of the Chao1, Goods coverage, observed species, and Shannon and Simpson indices (*P* = 0.452, 0.343, 0.179, 0.110, and 0.071, respectively). However, the acute exacerbation group showed significant differences in these indices compared to any of the remaining groups (*P* < 0.05). The results are summarized in Table 2.

**Table 2:** Alpha diversities of the three groups

Group	Chao1	Goods coverage	Observed species	Shannon	Simpson
B	90.30403*	0.989453*	61.8*	2.796442*	0.672084*
E	71.56429**	0.990647**	50.6**	2.753378**	0.738157**
R	85.14293	0.992488	71.1	3.74213	0.836411
F	10.068	11.754	11.661	11.102	10.435
*P*	0.000	0.000	0.000	0.000	0.000

Note: *P < 0.001, B vs E; **P = 0.001, E vs R. B, baseline; E, exacerbation; R, recovery.

**Figure 4:**
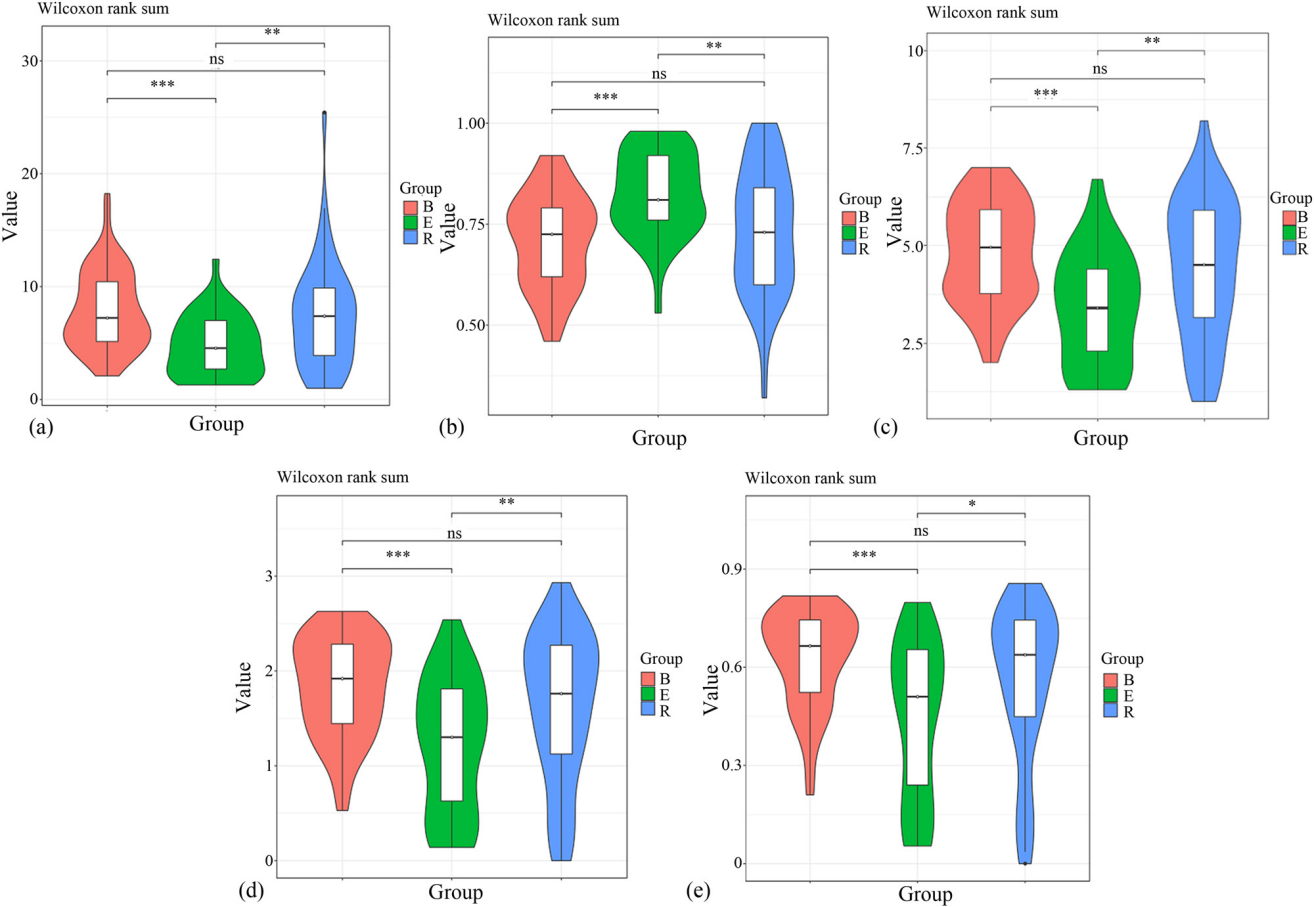
Violin plots of the alpha diversities of the three groups. (a) Chao1 violin. (b) Goods coverage violin. (c) Observed species violin. (d) Shannon violin. (e) Simpson violin. *P < 0.05, **P < 0.01, and ***P < 0.001, ns, no significant difference.

Violin analyses showed that the alpha diversity of the acute exacerbation group was significantly different from that of the remaining groups, particularly the baseline group. No significant difference was observed between the baseline and recovery groups (Figure 4).

### 3.3 Principal component analysis (PCA) and nonmetric multidimensional scaling (NMDS) analysis of beta diversity

**Figure 5:**
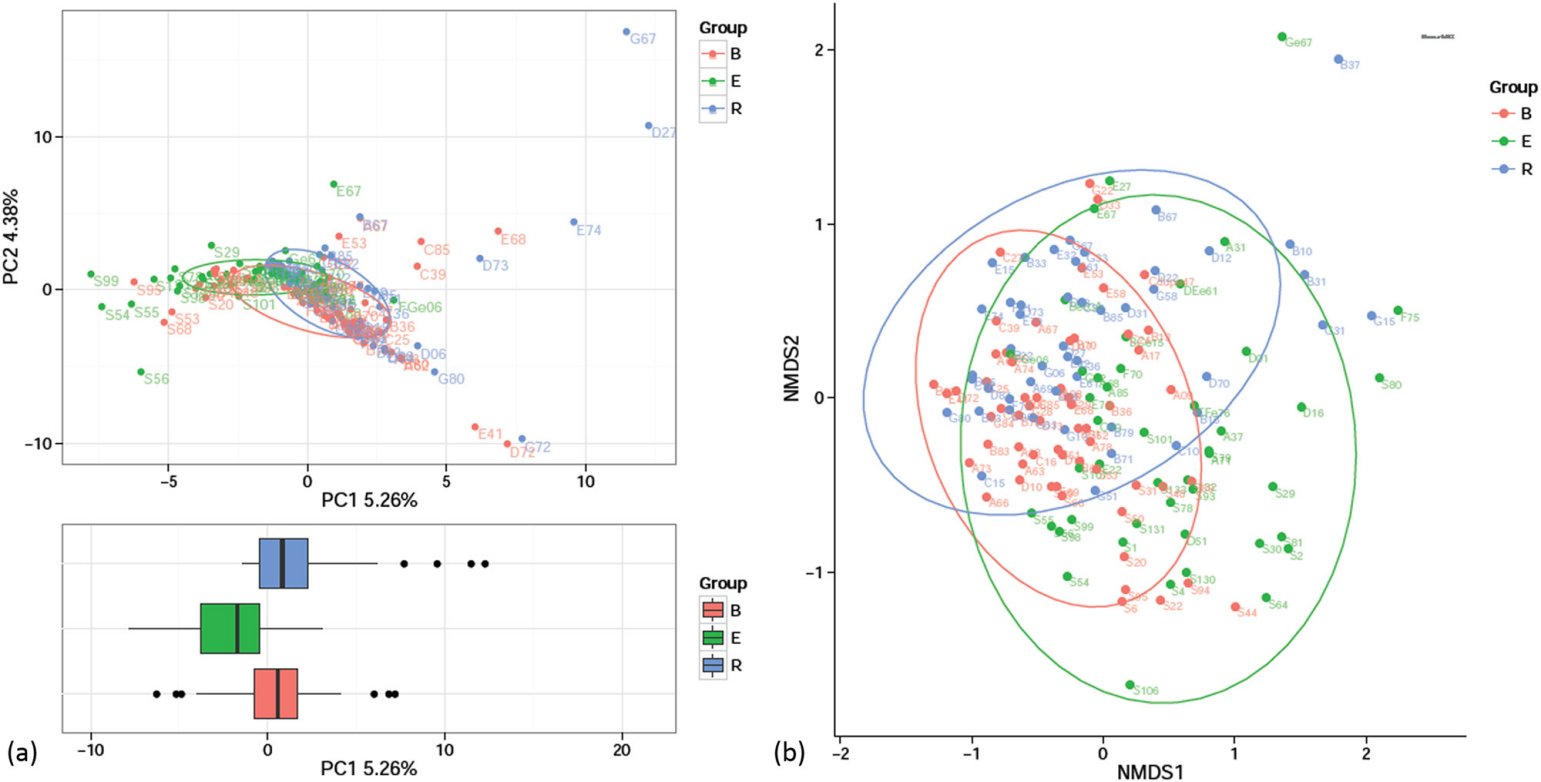
Analysis of the beta diversities of the three groups. (a) PCA: the axes indicate two or three eigenvalues that maximally reflect the variances. (b) NMDS analysis: the horizontal axis (NMDS1) and the longitudinal axis (NMDS2) are two ordering axes. In both panels, each point represents one sample, and the points of the same color indicate the same group. Similar samples were gathered together, and two samples with a large difference were distant from each other.

In this study, all the samples were from patients with nCFB, and therefore, the sample compositions of the three groups were similar. Nevertheless, the acute exacerbation group exhibited a large difference compared with the remaining groups, whereas the difference between the baseline and recovery groups was noticeable (Figure 5).

### 3.4 Multivariable analysis of the microorganisms

Adonis (i.e., PERMANOVA) analysis and ANOSIM were used to test possible significant differences among the groups.

#### 3.4.1 Differences among the groups

The three groups showed 30 significantly differentially abundant OTUs. At the phylum level, there were seven OTUs with significantly differential abundances, including Firmicutes, Proteus, Bacteroides, Actinomycetes, Fusobacteria, Tenericutes, and Spirochetes (the dominant flora constituents were mainly included in the first five phyla). At the genus level, the number of OTUs with a significantly differential abundance was 27. However, due to a partial lack of raw data, an analysis at the species level was not completed (Table 3).

**Table 3:** OTUs with significantly differential abundances at different levels

Method	OTU	Phylum	Class	Order	Family	Genus	Species
ANOVA	30	7	13	17	26	27	8
Kruskal–Wallis	52	8	18	24	33	47	20

#### 3.4.2 Differentially abundant genera

**Figure 6:**
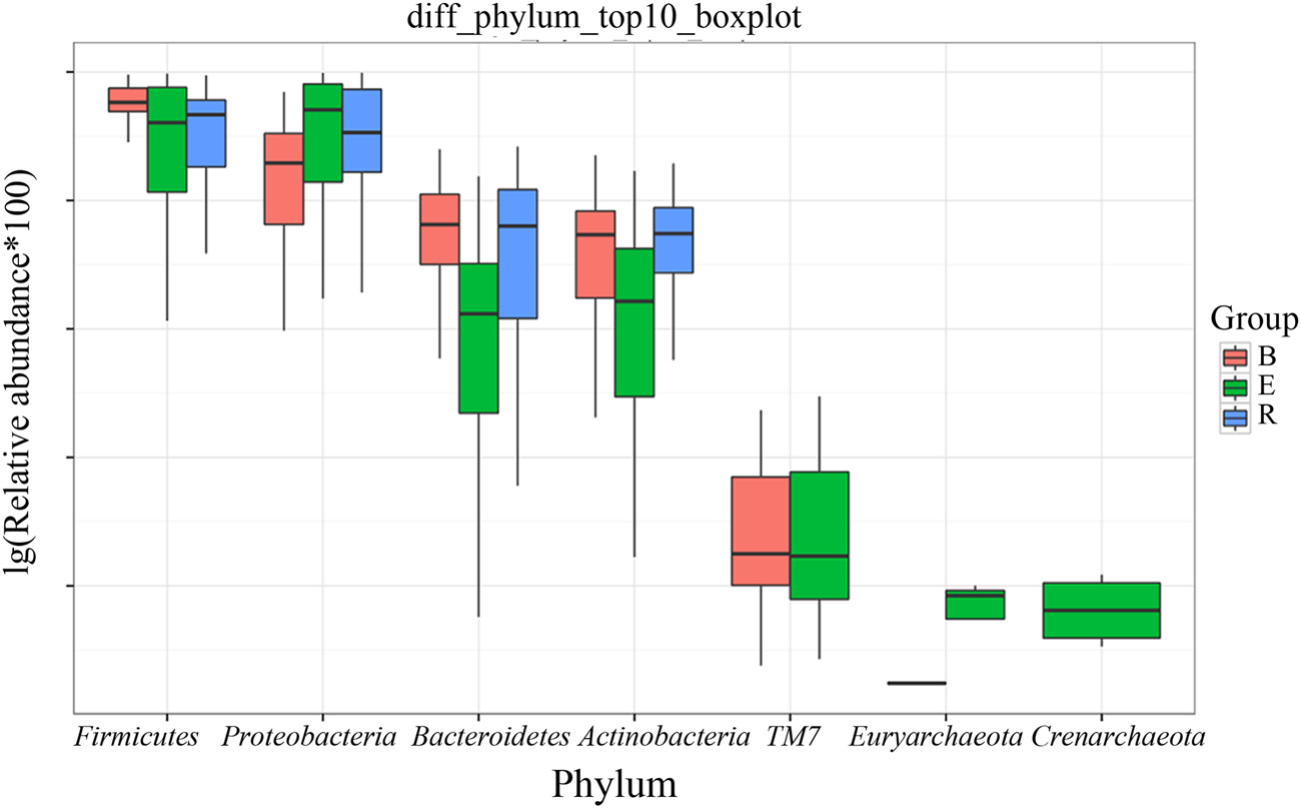
Boxplot of the top 7 phyla among the three groups. B, baseline; E, exacerbation; R, recovery.

**Figure 7:**
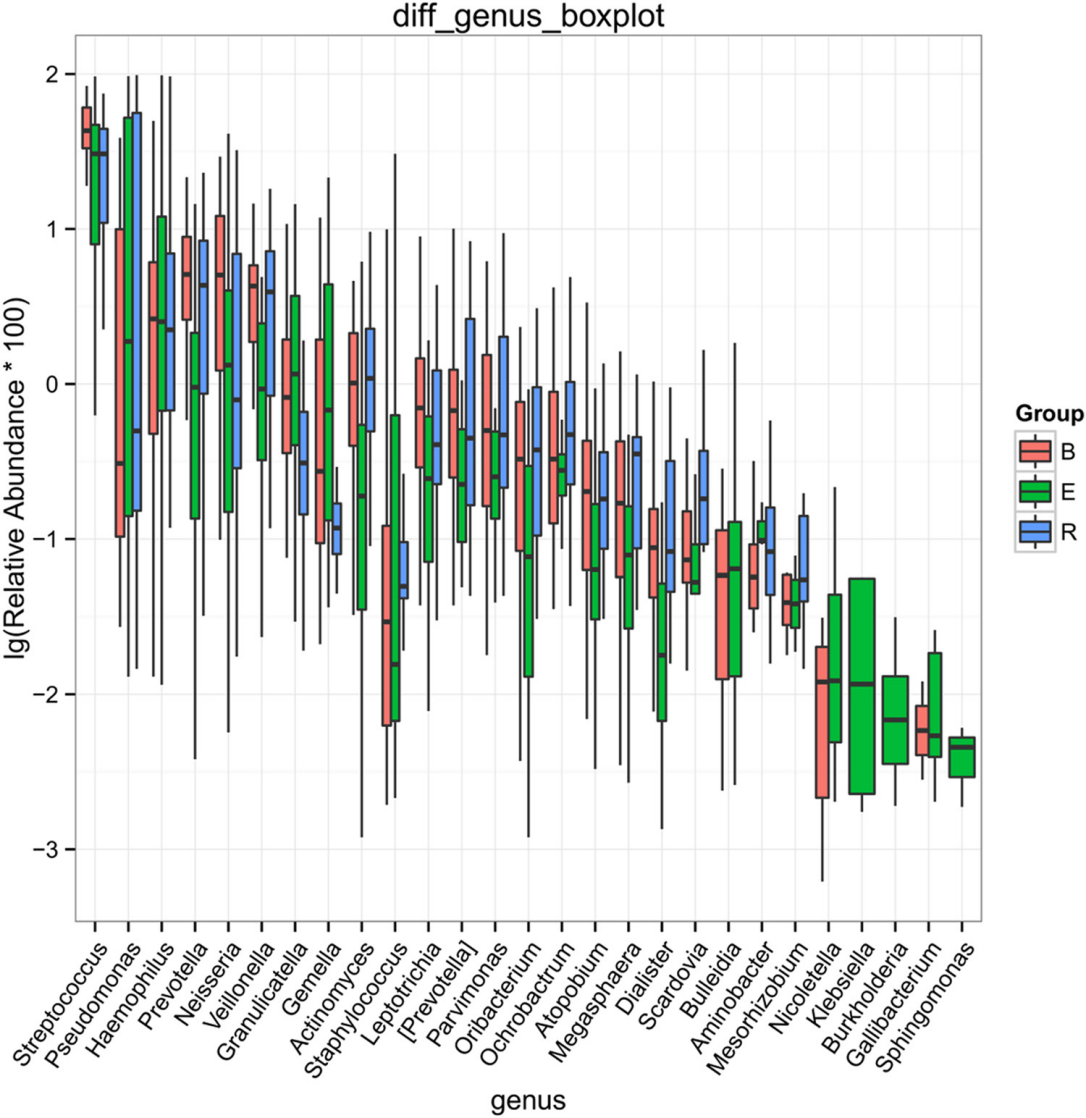
Boxplot of the differentially abundant genera (top 10) among the three groups. B, baseline; E, exacerbation; R, recovery.

**Figure 8:**
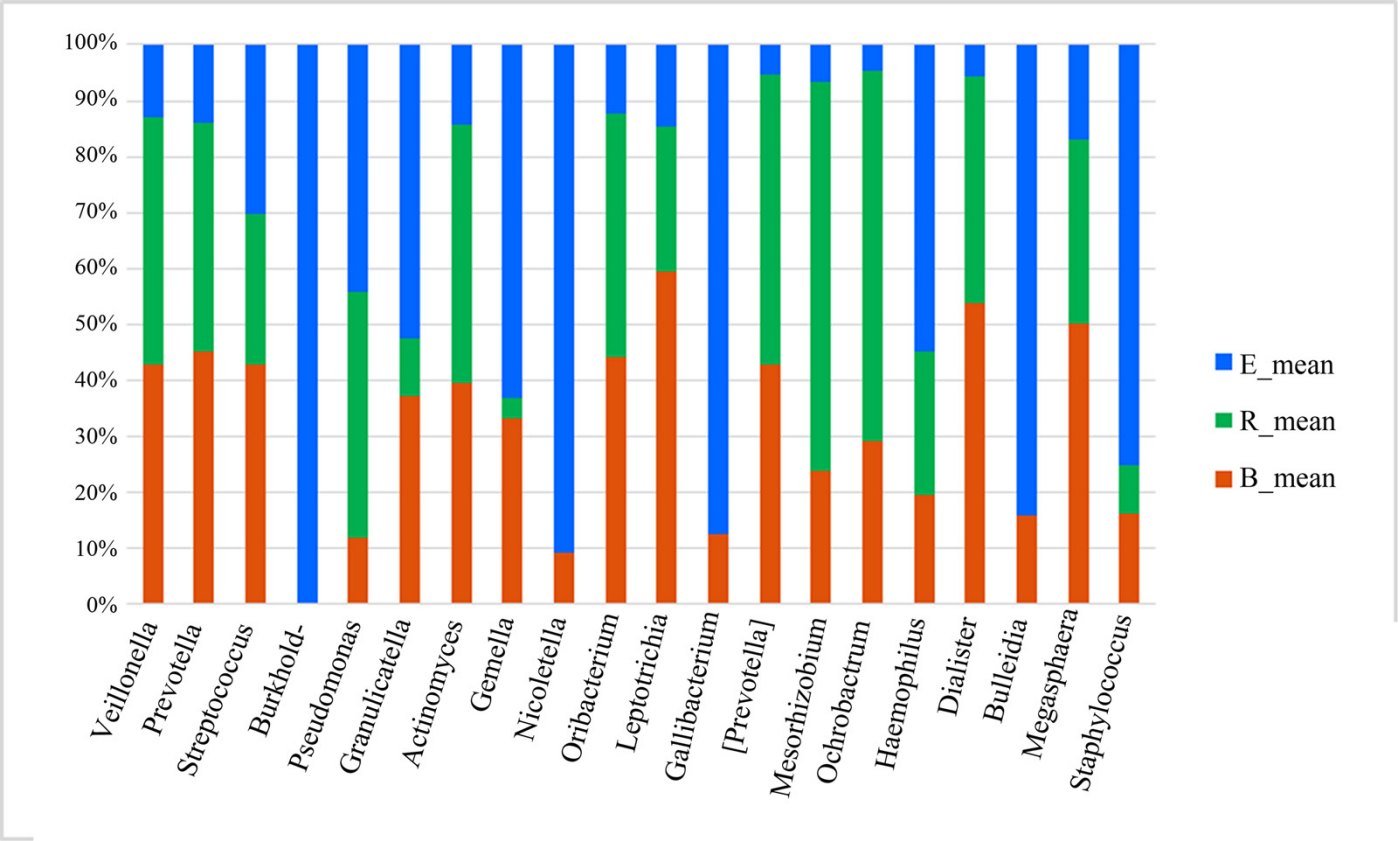
Stacked histogram of the differentially abundant genera among the three groups (percentage). B, baseline; E, exacerbation; R, recovery.

After analyzing the differentially abundant taxa at different levels, we selected the taxa with the top seven abundances for boxplot analysis to obtain the intragroup abundance and intergroup comparison outcomes of the dominant differentially abundant taxa. The three groups exhibited significant differences in the abundances and diversities of the phyla Bacteroides (*P* = 1.66 × 10^−05^), Firmicutes (*P* = 0.0003899), and Actinomycetes (*P* = 0.004875), and increased abundances and diversities of Proteus (*P* = 2.12 × 10^−05^), Crenarchaeota (*P* = 0.02058), Euryarchaeota (*P* = 0.022601), and TM7 (*P* = 0.02594) (Table 4). At the genus level, the acute exacerbation group exhibited noticeably decreased abundances and diversities of Burkholderia, Prevotella, Oribacterium, Neisseria, Veillonella, Leptotrichia, Actinomycetes, Mesorhizobium, Bacteroides, Megacocci, Mirabilis, Scardovia, Aminobacilli, and Micromonospora compared with those in the remaining groups. The abundances and diversities of Burkholderia, Pseudomonas, *Streptococcus granulosus*, Nicoletella, Haemophilus, Bulletidia, and Staphylococcus noticeably increased compared with those in the baseline group. The findings at the phylum and genus levels in the recovery group were comparable to those in the baseline group, but the mean abundance value of the genus Pseudomonas (0.246435) was closer to that of the acute exacerbation group (0.247582). It is noteworthy that the average abundances of TM7 and Guangarchaea in the recovery group were zero and that the average abundances of Lactobacillus, Ochrobactrum, Aminobacter, Rhodococcus, and Achromobacter in the recovery group were significantly higher than those in the baseline group (*P <* 0.001; Figures 6–8).

**Table 4:** ANOVA of the abundances and diversities of the microorganisms at the phylum level

	Bacteroidetes	Proteobacteria	Firmicutes	Actinobacteria	Crenarchaeota	TM7	Euryarchaeota
B-Mean	0.07817288	0.223382312	0.6080541	0.06078536	0	8.90 × 10^−05^	5.81 × 10^−07^
R-Mean	0.072189358	0.426044182	0.4196319	0.06051731	0	0	0
E-Mean	0.025787458	0.478334768	0.4476249	0.03230325	6.61 × 10^−06^	0.000212	6.38 × 10^−06^
T-Statistic	11.86259313	11.57698109	8.2779996	5.51846275	3.986332099	3.742807	3.740274967
*P*	1.66 × 10^−05^	2.12 × 10^−05^	0.0003899	0.00487497	0.020581964	0.025944	0.02600638
Bonferroni_P	0.000248583	0.000318163	0.0058489	0.07312458	0.308729457	0.389156	0.390095695

Note: B, baseline; E, exacerbation; R, recovery.

#### 3.4.3 LEfSe analysis

**Figure 9:**
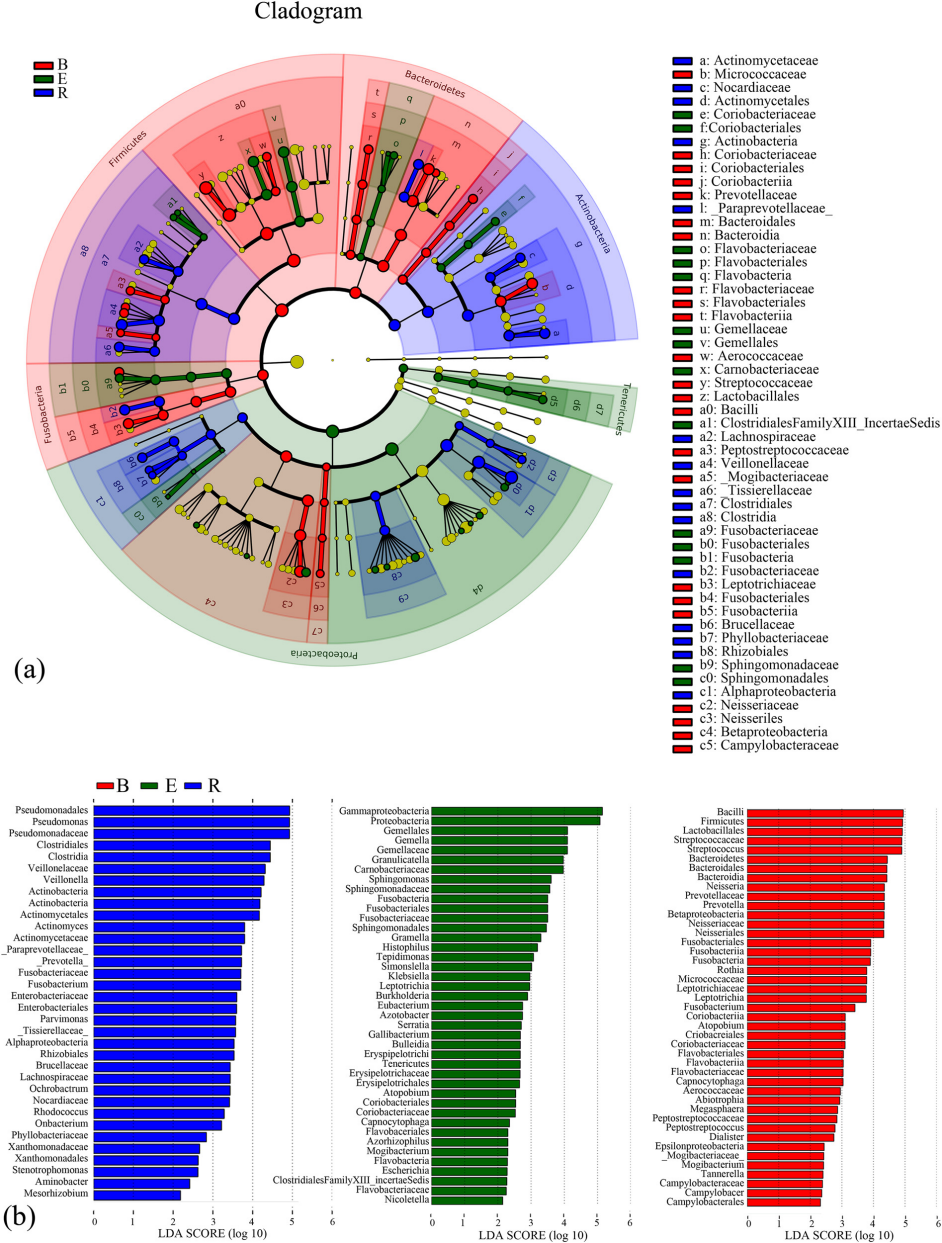
LEfSe analysis of the samples of the three groups. (a) Cladogram of the differentially abundant taxa. Different colors indicate different groups. Red nodes represent the differentially abundant taxa with a relatively high abundance in the baseline group, and green nodes represent the differentially abundant taxa with a relatively high abundance in the acute exacerbation group. Yellow nodes represent the taxa without significant differences between the baseline and acute exacerbation groups. The diameter of the node is positively proportional to the relative abundance of the taxon. The layers of nodes from inside to outside represent the phylum, class, order, family, and genus levels. The letters in the cladogram that are used to designate the taxa are defined in the right column. (b) Score graph of the differentially abundant taxa. Different colors indicate different groups, and the length of the bar indicates the relative abundance of the taxon. B, baseline; E, exacerbation; R, recovery.

The compositions, annotations, and contributions of the differentially abundant taxa among the three groups and the relative abundances of the differentially abundant taxa in each sample were analyzed. The baseline group exhibited the highest abundance, uniformity, and diversity. The recovery group showed higher uniformity than the acute exacerbation group; however, its abundance and diversity were lower than those of the latter group. Bacillus, Firmicutes, Lactobacillaceae, Streptococcaceae, and Streptococcus had the highest abundances in the baseline group, whereas Epsilonproteobacteria, Mogibacteriaceae, Mogibacterium, Tannerella, Campylobacteraceae, Campylobacter, and Campylobacterales had the lowest abundances. Gammaproteobacteria and Proteobacteria had the highest abundances in the acute exacerbation group, and Capnocytophaga, Flavobacteriales, Azorhizophilus, Mogibacterium, Flavobacteria, Escherichia, Clostridiales FamilyXIII_Incertae Sedis, Flavobacteriaceae, and Nicoletella had the lowest abundances. Pseudomonadales, Pseudomonas, and Pseudomonadaceae had the highest abundances in the recovery group, while Xanthomonadaceae, Xanthomonadales, Stenotrophomonas, Aminobacter, and Mesorhizobium had the lowest abundances (Figure 9).

#### 3.4.4 Adonis analysis

The total variance was decomposed based on the matrix (based on Bray–Curtis distance), and the interpretability of the grouping factor for the differences among the samples was analyzed. The statistical significance analysis mentioned above was performed using the permutation test (Table 5). The results showed that the grouping method in this study had statistical significance (*P =* 0.001): patients at different stages of nCFB exhibited differences in microbial flora structure and the abundances and diversity of the microorganisms at the acute exacerbation phase differed from those at the baseline phase and the recovery phase. However, as all the samples in this study were from patients with the same disease, the interpretability of this grouping method was low regarding the differences in intrapulmonary microorganisms at different phases of nCFB.

**Table 5:** Outcomes of the PERMANOVA/Adonis analysis (Bray–Curtis)

	D_f_	SumsOfSqs	MeanSqs	*F*. model	*R* ^2^	Pr (>F)	Signif
Group_factor	2	3.097	1.54872	7.3535	0.08984	0.001	***
Residuals	149	31.381	0.21061		0.91016		
Total	151	34.478			1		

*** indicates that the involved groups had a statistically significant difference.

#### 3.4.5 Correlation analysis

**Figure 10:**
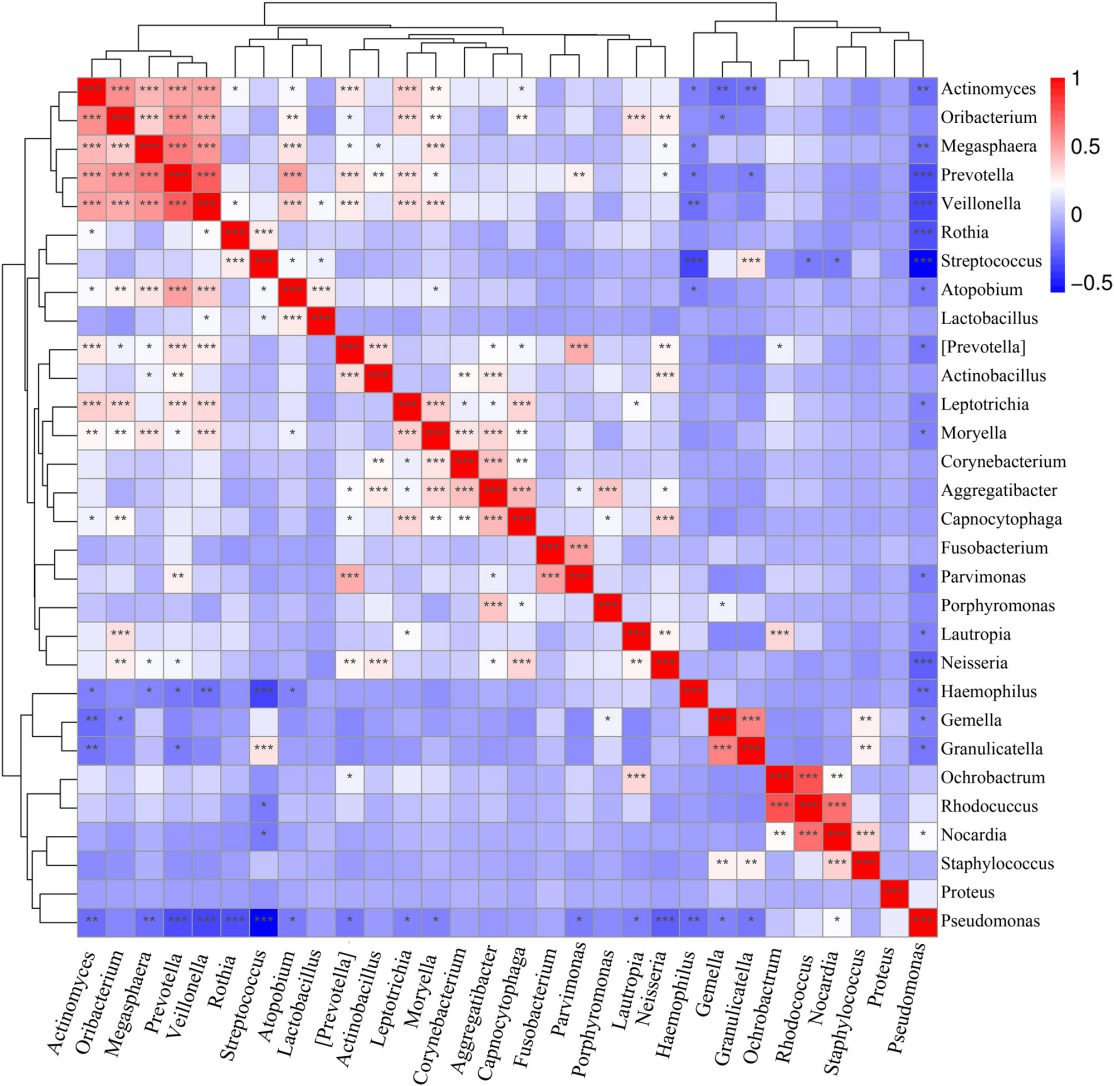
Heatmap of correlations. Note: Red denotes a positive correlation, whereas blue denotes a negative correlation. A larger number of asterisks indicate a higher correlation.

According to the heatmap of the correlation analysis results (Figure 10), Streptococcus had a considerable competitive relationship with Pseudomonas (*r* = −0.577 and *P* = 1.15 × 10^−13^) and Haemophilus (*r* = −0.363 and *P* = 6.4 × 10^−0.5^), and Rhodococcus and Nocardia had certain suppressive effects on Streptococcus. Rothia, Granulicatella, and Atopobium might be accomplices of Streptococcus, whereas the genus Pseudomonas was a loner. Except for being slightly intimate with Nocardia, Pseudomonas was “unfriendly” to Haemophilus (*r* = −0.241 and *P* = 0.0028), Prevotella (*r* = −0.334 and *P* = 2.66 × 10^−05^), Neisseria (*r* = −0.276 and *P* = 0.0006), Veillonella (*r* = −0.358 and *P* = 5.98 × 10^−06^), Rothia (*r* = −328 and *P* = 3.60 × 10^−05^), Actinomyces (*r* = −0.230 and *P* = 0.004), Megasphaera (*r* = −0.227 and *P* = 0.005), Granulicatella (*r* = −0.208 and *P* = 0.010), Gemella (*r* = −0.166 and *P* = 0.041), Parvimonas (*r* = −0.187 and *P* = 0.021), Lautropia (*r* = 0.182 and *P* = 0.025), and Leptotrichia (*r* = −0.169 and *P* = 0.037). Haemophilus was also antagonistic: it showed competitiveness with Prevotella, Veillonella, Actinomyces, Atopobium, and Megasphaera but not Pseudomonas. In contrast, Prevotella had a number of partners. It had synergetic relationships with Veillonella (*r* = 0.705 and *P* = 3.67 × 10^−24^), Megasphaera (*r* = 0.617 and *P* = 2.76 × 10^−17^), Oribacterium (*r* = 0.546 and *P* = 3.30 × 10^−13^), and Atopobium (*r* = 0.502 and *P* = 4.57 × 10^−11^) and was also a “friend” of Leptotrichia (*r* = 0.304 and *P* < 0.001), Parvimonas (*r* = 0.254 and *P* = 0.002), Actinobacillus (*r* = 0.224 and *P* = 0.005), and Moryella (*r* = 0.194 and *P* = 0.017). Neisseria had synergetic relationships with Capnocytophaga (*r* = 0.336 and *P* = 2.29 × 10^−05^), Oribacterium (*r* = 0.259 and *P* = 0.001), Lautropia (*r* = 0.246 and *P* = 0.002), Aggregatibacter (*r* = 0.202 and *P* = 0.013), and Megasphaera (*r* = 0.193 and *P* = 0.017). Veillonella was positively correlated with Megasphaera (*r* = 0.537 and *P* = 1.02 × 10^−12^), Actinomyces (*r* = 0.501 and *P* = 4.93 × 10^−11^), Oribacterium (*r* = 0.469 and *P* = 1.14 × 10^−09^), Atopobium (*r* = 0.369 and *P* = 3.00 × 10^−06^), Leptotrichia (*r* = 0.328 and *P* = 3.63 × 10^−05^), and Moryella (*r* = 0.306 and *P* = 0.000). Granulicatella was positively correlated with Gemella (*r* = 0.593 and *P* = 8.69 × 10^−16^) and Staphylococcus (0.250 and *P* = 0.002) but exhibited mutual suppression with Actinomyces (*r* = −0.215 and *P* = 0.008) and Streptococcus (*r* = 0.290 and *P* = 0.000).

## 4 Discussion

The mutual effect among microbial flora constituents in nCFB impacts the diversity and abundance of the microorganisms. This impact leads to changes in patients’ antibiotic sensitivity, pulmonary immunomodulatory capacity, and pathological conditions, which further influence the disease progression [19,20]. In chronic obstructive pulmonary disease (COPD), decreased abundances of Firmicutes and Proteobacteria have been reported [21,22,23]. With disease progression, nCFB can also gradually become complicated with COPD, which may contribute to the changes in pulmonary microecology in nCFB patients as well as nCFB patients’ progressive decline of pulmonary function. In this study, we performed an informatic analysis of the pulmonary microecology in patients with nCFB at different phases. We found that at the acute exacerbation stage, Firmicutes, Actinobacteria, and Bacteroidetes abundances decreased, while Proteobacteria and Archaea abundances increased, although these changes may not be the only factor altering the progression or prognosis of the disease.

In patients with nCFB at the acute exacerbation phase, the bacterial load does not change significantly compared to patients at other phases [13,14,15]. In patients with asthma and those with COPD, the dominant genera in healthy volunteers, such as Prevotella and Veillonella, exhibit low abundances [24,25,26]. Our study found that the abundance of Prevotella and Veillonella in patients with nCFB at the acute exacerbation phase noticeably decreased compared with those in patients in the remaining groups. Furthermore, we found that the nCFB patients at the acute exacerbation phase did not show a noticeable difference in bacterial load when compared to patients in any of the remaining groups. These results suggest that the pulmonary microecology of nCFB patients is in a state of dynamic equilibrium according to different disease stages. In addition, according to the literature [24,25,26], in patients with asthma and those with COPD, the abundances of *Pseudomonas aeruginosa* and *Haemophilus influenzae* increased, and that of *Staphylococcus aureus* also showed abnormalities. Although our results were consistent with those reported, we did not find abnormalities in the abundances of these taxa in nCFB patients at the acute exacerbation phase compared with those at other phases. The reason for this difference may be attributed to the different populations included in our study and in theirs. Compared with those in the baseline group, the abundance, uniformity, and diversity of the pulmonary microecology in the acute exacerbation group decreased. The recovery group exhibited the lowest abundance and diversity but the best uniformity among the three groups. This phenomenon may be attributed to antibiotic use or the interaction among bacterial genera. The possibility of self-adjusting the pulmonary microecology after insult should also not be excluded.

To date, most related studies have focused on the variations in Firmicutes and Proteobacteria abundances [21,22,23], and according to these studies, Firmicutes abundance in the pulmonary microecology of patients with COPD and those with asthma decreased significantly, whereas Proteobacteria abundance significantly increased. Similar changes in the pulmonary microecology were observed in nCFB patients at the acute exacerbation stage in this study. In addition, this study also revealed that the abundance and diversity of Bacteroidetes and Actinomycetes in these patients were lower than those in the baseline and recovery groups. These changes were accompanied by the increased abundance and diversity of Proteobacteria, Crenarchaeota, Euryarchaeota, and TM7. Normally, Crenarchaeota and Euryarchaeota live in extreme environments [27], which resemble the original environment of life. Is it reasonable to assume that the pulmonary environment in the acute exacerbation stage aggravates into a stage of destruction, considering that most Crenarchaeota and Euryarchaeota bacteria are anaerobic? With the removal of a large quantity of sputum, the suppressive effect of antibiotics on pathogenic bacteria, ciliary function, the immune system, and the environment of the lower respiratory tract is improved. Therefore, will the environment at the recovery stage no longer be suitable for Crenarchaeota and Euryarchaeota, which then migrate or evolve? Although the correlation analysis in this study did not reveal a correlation of increased Crenarchaeota and Euryarchaeota abundances with increased Proteobacteria abundance, the change in Crenarchaeota and Euryarchaeota abundances discovered in this study may provide a new idea for related research to be conducted in the future.

The interactions among bacterial genera are extremely subtle. According to previous studies [30,31], the abundances of Prevotella and Veillonella significantly increase in the pulmonary microecology of patients with lung cancer and those with asthma [28,29]. This study found that Prevotella and Veillonella cooperated with each other and that Ochrobactrum and Rhodococcus were synergetic. Pseudomonas is an independent dominant bacterial genus, and it exerted noticeable suppressive effects on Haemophilus, Prevotella, Neisseria, Veillonella, Rothia, Actinomyces, Leptotrichia, Parvimonas, Capnocytophaga, Atopobium, Lautropia, and Streptococcus. The interrelations among the bacteria suggest a hypothesis: in addition to the use of drugs to suppress or kill pathogenic bacteria, it means to increase the abundance of the competitor of the pathogenic bacteria, such as bacterial transplantation and regulation of the intestinal flora, may also help enhance the treatment effect. Oral administration or transplantation of probiotics can improve pulmonary resistance against viruses, *Mycobacterium tuberculosis*, and other pathogenic bacteria [30,31,32,33,34]. Currently, broad- or ultrabroad-spectrum antibiotics have been used to target *Pseudomonas aeruginosa* in clinical practice [35,36,37,38]. However, with these antibiotics, *P. aeruginosa* has become increasingly drug-resistant, while its competitor bacteria have become increasingly less drug-resistant. This cycle repeats, and consequently, *P. aeruginosa* cannot be brought under satisfactory control. The mutual competitiveness among bacteria may provide us with a new idea in clinical practice: can we increase the abundance and activity of the competitor bacteria of *P. aeruginosa* to suppress its activity?

This study had some limitations. First, because the pulmonary microecological characteristics of healthy individuals in the same analysis region could not be obtained in the database, a comparative analysis of pulmonary microecology between healthy individuals and patients with bronchiectasis could not be performed. Second, due to the partial absence of the original data, we could not perform analysis of bacterial species such as *P. aeruginosa*, *Pseudomonas mucilaginosa,* and *Staphylococcus aureus*, which play key roles in the prognosis of nCFB. Therefore, the guiding significance of this study regarding antibiotic determination and immunoregulation is compromised. Third, all the data used in this study were from the outcomes of 16S rRNA-based sputum analysis, which might suffer from bias due to the influence of the upper respiratory tract. If bronchoalveolar lavage fluid samples had been obtained for next-generation sequence-based longitudinal analysis, the results of this study would be more persuasive. Fourth, our analysis was limited to pulmonary microorganisms at different stages of nCFB. Currently, whole-genome detection techniques for the intestines have advanced and have already been applied to detect intestinal diseases [39,40]. There exists comorbidity between multiple pulmonary diseases and intestinal diseases, which is associated with the intermigration and disturbance of microbes [41]. In this study, however, we did not take the interrelation between pulmonary microecology and intestinal microecology into consideration. What role does this intermigration and disturbance mechanism play in nCFB and whether we can take advantage of this disturbance effect to improve the flora structure in nCFB by regulating the intestinal microecology and thus improve patients’ conditions remains to be investigated. Fifth, this study was originally planned as bioinformatics analysis and did not involve experimental research. In addition, in the future, analysis of lower respiratory tract samples from nCFB patients can be combined with the exploration of the mechanism underlying the impact of the changes in intestinal microorganisms on the immunoregulation of the gut-lung axis, which can be expected to reveal more nonantibiotic therapies for nCFB.

There were changes in the abundance and diversity of the bacterial flora at the acute exacerbation stage of nCFB. Proteobacteria may be a biological signal of nCFB changes, and the appearance of Crenarchaeota and Euryarchaeota may be a biological signal of disease aggravation. The mutual competitiveness and cooperation among bacteria may provide new ideas for clinical medication in the treatment of nCFB.
